# Association of volatile organic compound levels with chronic obstructive pulmonary diseases in NHANES 2013–2016

**DOI:** 10.1038/s41598-024-67210-7

**Published:** 2024-07-12

**Authors:** Xiangliang Liu, Yu Chang, Chengyao Xu, Yuguang Li, Yao Wang, Yao Sun, Meilin Duan, Wei Li, Jiuwei Cui

**Affiliations:** 1 The First Hospital of Jilin University, No.1 Xinmin Street, Changchun, 130012 China; 2 Jilin Provincial Institute for Drug Control, Changchun, 130022 China

**Keywords:** Volatile organic compounds (VOCs), Chronic obstructive pulmonary diseases (COPD), The National Health and Nutrition Examination Survey (NHANES), Restricted cubic splines (RCS), Weighted quantile sum (WQS) regression, Machine learning algorithms, Chronic obstructive pulmonary disease, Environmental monitoring

## Abstract

Volatile organic compounds (VOCs) represent a significant component of air pollution. However, studies evaluating the impact of VOC exposure on chronic obstructive pulmonary disease (COPD) have predominantly focused on single pollutant models. This study aims to comprehensively assess the relationship between multiple VOC exposures and COPD. A large cross-sectional study was conducted on 4983 participants from the National Health and Nutrition Examination Survey. Four models, including weighted logistic regression, restricted cubic splines (RCS), weighted quantile sum regression (WQS), and the dual-pollution model, were used to explore the association between blood VOC levels and the prevalence of COPD in the U.S. general population. Additionally, six machine learning algorithms were employed to develop a predictive model for COPD risk, with the model’s predictive capacity assessed using the area under the curve (AUC) indices. Elevated blood concentrations of benzene, toluene, ortho-xylene, and para-xylene were significantly associated with the incidence of COPD. RCS analysis further revealed a non-linear and non-monotonic relationship between blood levels of toluene and m-p-xylene with COPD prevalence. WQS regression indicated that different VOCs had varying effects on COPD, with benzene and ortho-xylene having the greatest weights. Among the six models, the Extreme Gradient Boosting (XGBoost) model demonstrated the strongest predictive power, with an AUC value of 0.781. Increased blood concentrations of benzene and toluene are significantly correlated with a higher prevalence of COPD in the U.S. population, demonstrating a non-linear relationship. Exposure to environmental VOCs may represent a new risk factor in the etiology of COPD.

## Introduction

Chronic obstructive pulmonary disease (COPD) is a common, preventable, and treatable disease characterized by persistent respiratory symptoms and airflow limitation, resulting from airway and/or alveolar abnormalities usually caused by significant exposure to noxious particles or gases^1^. COPD is a major public health problem, affecting more than 5% of the population and associated with high morbidity and mortality worldwide^2^. Cigarette smoking is recognized as the most important risk factor for COPD. However, only 15–20% of smokers develop clinically significant COPD, suggesting that other environmental exposures, such as air pollution, likely contribute to the development and progression of COPD^3^.

Volatile organic compounds (VOCs) are an important component of air pollution and some VOCs such as benzene and toluene have been associated with COPD exacerbations^4,5^. However, previous studies evaluating the effects of VOC exposure on COPD have mostly focused on single pollutant models. Given that the general population is usually exposed to multiple VOCs simultaneously, it is crucial to understand the cumulative health effects of VOCs mixtures. As a subclass of artificial intelligence, machine learning is believed to be capable of handling larger scale and higher dimensional data, with a higher accuracy rate in terms of diagnosis and prognosis. In this cross-sectional study based on the National Health and Nutrition Examination Survey (NHANES), we aimed to investigate the association between blood VOCs levels and COPD prevalence in US adults. We specifically focused on examining the dose–response relationships and cumulative effects of VOC exposure using flexible nonlinear models and weighted quantile sum (WQS) regression. Additionally, we constructed several machine learning models to predict the incidence of COPD and evaluated the performance of the optimal model. We also conducted a dual-pollution model analysis to evaluate the correlation between VOC levels and COPD. The findings from this study will provide novel insights into the link between environmental VOCs exposure and COPD development. The results may also inform environmental health policies aimed at better controlling VOC emissions and protecting susceptible populations from COPD.

## Methods

### Study population

NHANES is a comprehensive survey project initiated by the Centers for Disease Control and Prevention (CDC) and maintained by the National Center for Health Statistics (NCHS), aimed at collecting and analyzing health and nutrition data of the U.S. population. The primary objective of NHANES is to measure and monitor health indicators in the U.S. population, including dietary habits, nutritional intake, health status, chronic diseases, anthropometric measurements, and biochemical markers. In this analysis, we combined data from two survey cycles (2013–2014 and 2015–2016) involving 20,146 participants, all of whom provided informed consent. All methods were performed in accordance with relevant guidelines and regulations.

**Figure 1:**
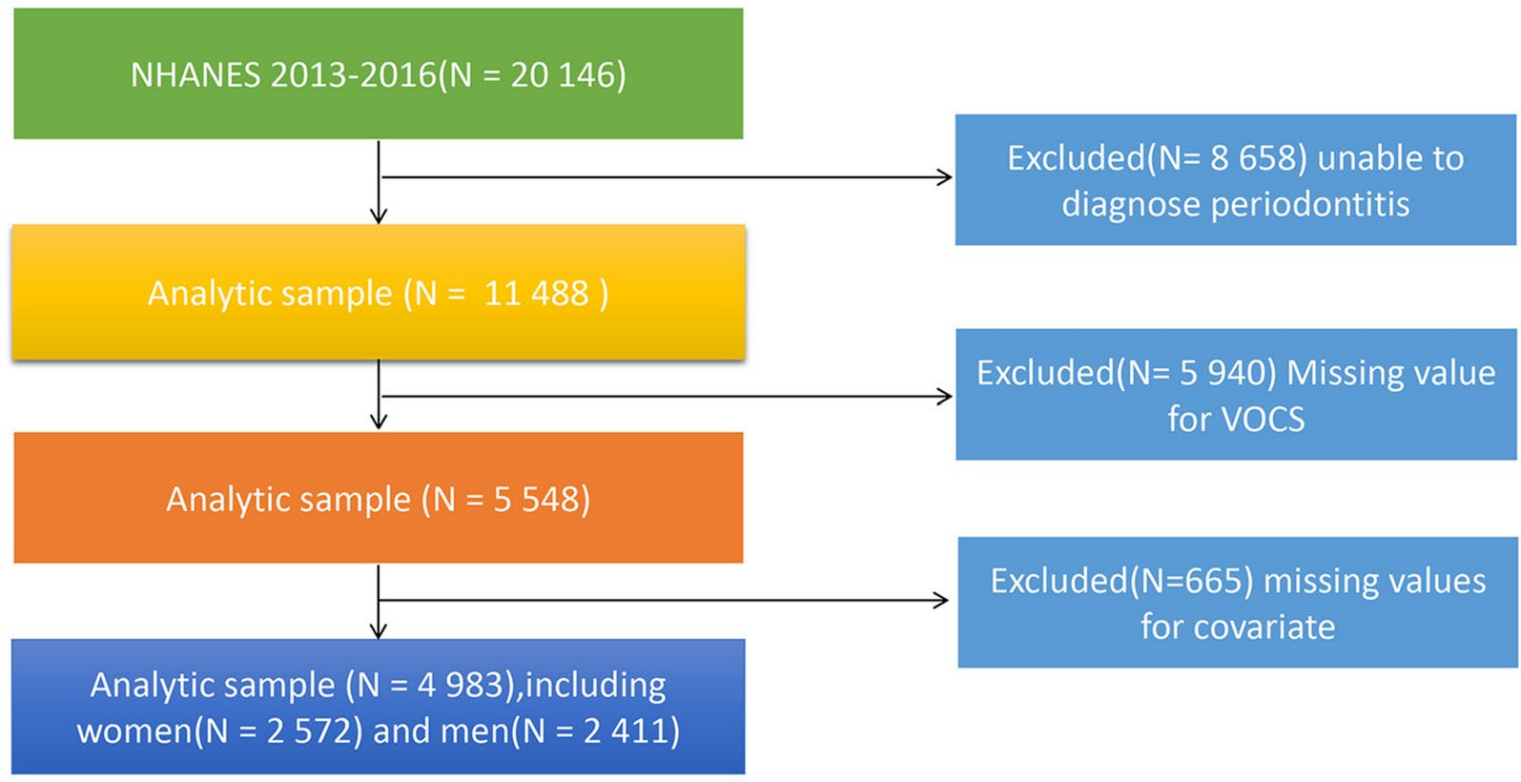
The flow diagram of the study participants, from NHANE 2013–2016.

A total of 8658 participants were excluded due to the absence of a chronic obstructive pulmonary disease diagnosis, and 5940 were excluded due to the lack of VOC data. An additional 665 participants were excluded due to missing data on other covariables. Ultimately, 4983 participants were included in this study, comprising 2411 males and 2572 females. Detailed information is presented in Fig. 1.

### Data collection

#### Assessment of VOCs

VOCs, commonly used as solvents, degreasers, and cleaners, represent a broad category of chemicals extensively utilized in industrial and consumer products. The measurement of VOCs primarily involves the quantitative determination of VOCs in household tap water and whole human blood. In domestic water, VOCs include trihalomethanes (THMs) such as chloroform, bromodichloromethane, dibromochloromethane, and bromoform^6,7^. The analysis employs headspace solid-phase microextraction (SPME) coupled with mass spectrometry (MS) and capillary gas chromatography (GC). Measurement of blood disinfection by-products (DBPs) such as chloroform, bromodichloromethane, dibromochloromethane, and bromoform, as well as methyl tertiary-butyl ether (MTBE), is conducted using GC combined with high-resolution MS. Quantification of trace levels of DBPs and MTBE in blood is performed using selected ion monitoring and isotope dilution techniques. Quantitative measurement of benzene, tetrachloroethylene, 1,4-dichlorobenzene, toluene, o-xylene, and p-xylene is conducted using SPME combined with gas chromatography methods^8^. In this study, we investigated several VOCs from the NHANES database that were detected above the limit of detection, including tetrachloroethene, benzene, 1,4-dichlorobenzene, o-xylene, toluene, m-p-xylene, chloroform, and bromodichloromethane, to explore their relationship with COPD.

#### Diagnosis of COPD

The diagnosis of COPD is primarily conducted through spirometry tests administered by trained technicians, with subsequent data interpretation and evaluation performed by professional pulmonologists. According to the spirometry guidelines established by the European Respiratory Society (ERS) and the American Thoracic Society (ATS), a forced expiratory volume in one second (FEV1) to forced vital capacity (FVC) ratio of less than 0.70 is indicative of COPD.

Furthermore, if participants affirmatively answer the question: “Have you been told by a doctor or other health professional that you have emphysema, chronic obstructive pulmonary disease (COPD), or chronic bronchitis?”, they are recorded as having COPD. Additionally, the use of selective phosphodiesterase-4 inhibitors, mast cell stabilizers, leukotriene modifiers, and inhaled corticosteroids as bronchodilators, as well as being over 40 years of age, having a history of smoking, or having chronic bronchitis, are considered criteria for the diagnosis of COPD^9^.

#### Covariates

Covariates included gender, age, race/ethnicity (Mexican American, Non-hispanic Black, Non-hispanic White, Other Hispanic, Other Race—Including Multi-Racial), educational level (low high school, high school, College or above), Body Mass Index (BMI) (< 25, [25, 30), ≥ 30), Poverty Income Ratio (PIR) (< 1, 1–3, ≥ 3) and drinking status (Former drinker (individuals who used to drink but have now stopped), Never drinker (individuals who have never consumed any alcoholic beverages), Mild drinker (individuals who consume between 1 and 2 standard drinks per day), Moderate drinker (individuals who consume up to 4 standard drinks per day for men, and up to 3 for women), Heavy drinker (individuals who consume more than 4 standard drinks per day for men, and more than 3 for women)).

### Statistical analysis

In our analysis, we applied the NHANES-recommended sample weights for each participant and employed the recommended weighting methods. To compare differences between the COPD group and the non-COPD group, we expressed continuous variables as weighted means (± standard deviation) and used weighted t-tests to describe statistical differences. Categorical variables were expressed as sample counts (weighted percentages), with weighted chi-square tests used to describe statistical differences. We categorized continuous VOCs concentrations by quartiles (Q1: < 25th percentile, Q2: 25–50th percentile, Q3: 50–75th percentile, Q4: 75–100th percentile); the specific concentration distributions of the 8 organic compounds are detailed in Table 1.

**Table 1:** The concentration distribution of various volatile organic compounds.

VOCs (urine, ng/ml)	Q1	Q2	Q3	Q4
Tetrachloroethene	0.0339–0.064	0.064–0.092	0.092–0.173	0.173–16
Benzene	0.017–0.043	0.043–0.092	0.092–0.205	0.205–2.75
1,4-Dichlorobenzene	0.028–0.069	0.069–0.143	0.143–0.483	0.483–115
oXylene	0.017–0.03	0.03–0.041	0.041–0.059	0.059–6.52
mpXylene	0.024–0.049	0.049–0.075	0.075–0.159	0.159–24.6
Toluene	0.0117–0.048	0.048–0.073	0.073–0.173	0.173–14.7
Chloroform	0.0057–0.011	0.011–0.016	0.016–0.0243	0.0243–0.594
Bromodichloromethane	0.004–0.007	0.007–0.009	0.009–0.013	0.013–0.068

We used weighted logistic regression models to assess the association between blood VOCs and COPD, estimating for the crude model, Model 1 (adjusted solely based on gender, age, and race), and Model 2 (which includes adjustments for PIR, BMI, educational level, and drinking status based on Model 1). We observed the statistical differences between Tetrachloroethene, Benzene, 1,4-Dichlorobenzene, O-Xylene, M-p-Xylene, Toluene, Chloroform, Bromodichloromethane and COPD, employing restricted cubic splines (RCS) to explore potential nonlinear relationships between blood levels of Benzene, Toluene, M-p-Xylene, O-Xylene and COPD. Furthermore, we constructed a WQS regression model to analyze the relationship between mixed exposure to organic compounds in VOCs and COPD, as well as the weighting of each compound^10^.

$$ {\text{g}}\left( {\upmu } \right) = \beta_{o} + \beta_{1} \left( {\mathop \sum \limits_{i = 0}^{c} \omega_{i} \varphi_{i} } \right) + z^{\prime } \Phi $$

$$ WQS = \mathop \sum \limits_{i = 1}^{c} \overline{\omega }_{i} \varphi_{i} $$

The basic weighted index model is as follows: ${\upbeta }_{{\text{o}}}$ represents the intercept, ${\upbeta }_{1}$ represents the regression coefficients, c denotes the number of organic compounds included in the analysis, ${\text{z}}^{\prime }$ and Φ represent the matrix and coefficients for covariates, ${\upomega }_{{\text{i}}}$ denotes the weighted indices, with each index ranging from 0 to 1 (0 ≤ ${\upomega }_{{\text{i}}}$ ≤ 1), summing up to a total of 1. $\varphi_{i}$ denotes the quartile for each organic compound concentration, with ($\varphi_{i}$ = 0,1,2,3) representing the 1st, 2nd, 3rd, or 4th quartile, respectively. $\left( {\mathop \sum \nolimits_{i = 0}^{c} \omega_{i} \varphi_{i} } \right)$ is the sum of weighted quartiles for c components. ${\mathbf{g}}\left( {{\varvec{\upmu}}} \right)$ represents any differentiable link function. We also assumed a linear function fitting a Gaussian distribution and randomly divided the data into a training set (60%) and a validation set (40%), estimating the weights of the 8 organic compounds in the training set^11^. Additionally, we utilized the dual-pollutant model to evaluate the association between the exposure to 8 VOCs and COPD, adjusting for BMI, age, PIR, race, and drinking status.

We have developed and constructed six machine learning models using VOCs and demographic data screened from the NHANES database. These include Logistic Regression (LR)^12^, Random Forest (RF)^13^, Extreme Gradient Boosting (XGBoost)^14^, Support Vector Machine (SVM)^15^, Decision Tree (DT)^16^, and Naive Bayes Classifier (NBC)^17^, to predict the incidence of COPD. The predictive ability of the six machine learning models was evaluated by drawing Receiver Operating Characteristic (ROC) curves and using the Area Under the Curve (AUC). We also described the Area Under the Curve (AUC), precision, accuracy, recall, and F1-Score of the best model.

All the aforementioned statistical analyses corresponding figures were performed using R Project for Statistical Computing (version 4.2.3), with all tests being two-sided and *p*-values < 0.05 considered to be significant.

### Ethical approval and informed consent

The NHANES agreement has been reviewed and approved by the National Center for Health Statistics Research Ethics Committee. All participants provided written informed consent before participating.

## Results

### Baseline characteristics

Participants were categorized based on the presence of COPD into the COPD group (1352 individuals) and the non-COPD group (2721 individuals). Participants in the COPD group were more likely to be under the age of 65, non-Hispanic whites, possessing at least a high school education, and former drinkers. Compared to the non-COPD group, the distribution of VOCs such as benzene and toluene in blood had statistical significance. There were no significant statistical differences in the exposure to VOCs such as tetrachloroethene, 1,4-Dichlorobenzene, o-xylene, m-xylene, chloroform, and bromodichloromethane in the blood. Statistical differences were observed for the aforementioned baseline characteristics. Specific details can be found in Table 2.

**Table 2:** Characteristics of participants by chronic obstructive pulmonary disease, NHANES 2013–2016.

	Level	No COPD	COPD	*P*-value
N (%)		2721 (66.81)	1352 (33.19)	
Sex (%)				0.4119
	Female	2487 (51.74)	85 (48.30)	
	Male	2320 (48.26)	91 (51.70)	
Age (%)				< 0.0001
	[30, 65)	3851 (80.11)	94 (53.41)	
	≥ 65	956 (19.89)	82 (46.59)	
Ethnicity (%)				< 0.0001
	Mexican American	708 (14.73)	12 (6.82)	
	Non-Hispanic Black	997 (20.74)	30 (17.05)	
	Non-Hispanic White	1850 (38.49)	106 (60.23)	
	Other Hispanic	528 (10.98)	17 (9.66)	
	Other race—including Multi-racial	724 (15.06)	11 (6.25)	
Education (%)				0.0043
	Low high school	1023 (21.28)	51 (28.98)	
	High school	1061 (22.07)	47 (26.70)	
	College or above	2723 (56.65)	78 (44.32)	
BMI (%)				0.3268
	< 25	1419 (29.52)	45 (25.57)	
	[25, 30)	1540 (32.04)	54 (30.68)	
	≥ 30	1848 (38.44)	77 (43.75)	
Poverty income ratio (%)				
	< 1	1074 (22.09)	58 (32.40)	0.0024
	[1, 3)	2057 (42.31)	73 (40.78)	
	≥ 3	1731 (35.60)	48 (26.82)	
Alcohol drinkers (%)				< 0.0001
	Former drinker	691 (15.75)	60 (36.14)	
	Heavy drinker	854 (19.47)	22 (13.25)	
	Mild drinker	1459 (33.26)	49 (29.52)	
	Moderate drinker	685 (15.62)	22 (13.25)	
	Never drinker	697 (15.89)	13 (7.83)	
Tetrachloroethene (mean (SD))		0.059 (0.378)	0.041 (0.048)	0.5614
Benzene (mean (SD))		0.063 (0.121)	0.103 (0.170)	0.0001
1,4-Dichlorobenzene (mean (SD))		0.844 (4.899)	1.148 (5.754)	0.4453
O-Xylene (mean (SD))		0.045 (0.262)	0.036 (0.048)	0.6744
Toluene (mean (SD))		0.188 (0.438)	0.263 (0.341)	0.0334
M-p-Xylene (mean (SD))		0.149 (0.938)	0.131 (0.190)	0.8116
Chloroform (mean (SD))		0.015 (0.022)	0.014 (0.012)	0.5187
Bromodichloromethane (mean (SD))		0.005 (0.004)	0.005 (0.003)	0.4947

*SD*, Standard deviation.

### Association between blood VOCs with COPD

The relationship between VOCs and the occurrence of COPD was assessed using logistic regression models. In the crude model, the risk of COPD in the highest quartile (Q4) for blood benzene concentration was 145% greater compared to the lowest quartile (Q1 group) (Q4: OR 2.45, 95%CI 1.54–3.77, *P* < 0.001). Compared to the Q1 group for blood o-xylene concentration, the risk of developing COPD in the Q4 group was increased by 2.37 times (Q4: OR 2.37, 95%CI 1.44–3.73, *P* < 0.001). The risk of COPD in the Q4 group for blood m-xylene was 1.99 times higher compared to the Q1 group (Q4: OR 1.99, 95%CI 1.34–2.91, *P* < 0.001). When comparing the Q4 group with the Q1 group for blood toluene, the increased risk of developing COPD was 1.67 times (Q4: OR 1.67, 95%CI 1.12–2.52, *P* = 0.013).

In Model 1, compared to the Q1group of blood benzene concentration, the risk of COPD was 2.36 times higher in the third quartile (Q3) (Q3: OR 2.36, 95%CI 1.40–3.83, *P* < 0.001), and 3.19 times higher in the Q4 group (Q4: OR 3.19, 95%CI 1.95–5.07, *P* < 0.001). Compared to the Q1 group for blood 1,4-dichlorobenzene concentration, the risk of developing COPD in the Q4 group was 1.76 times greater (Q4: OR 1.76, 95%CI 1.06–2.84, *P* = 0.023). The risk of COPD in the Q4 group compared to the Q1 group for blood m-xylene concentration was increased by 2.20 times (Q4: OR 2.20, 95%CI 1.46–3.29, *P* < 0.001). When comparing the Q4 group with the Q1 group for blood toluene concentration, the increased risk of developing COPD was 1.99 times (Q4: OR 1.99, 95%CI 1.31–3.05, *P* = 0.001). In Model 2, the risk of COPD increased by 2.04 times in the second quartile (Q2) compared to the Q1 group for blood benzene concentration (Q2: OR 2.04, 95%CI 1.14–3.49, *P* = 0.012), by 2.03 times in the Q3 group (Q3: OR 2.03, 95%CI 1.16–3.41, *P* = 0.010), and by 2.70 times in the Q4 group (Q4: OR 2.70, 95%CI 1.58–4.49, *P* < 0.001). Compared to the Q1 group for blood 1,4-dichlorobenzene concentration, the risk of developing COPD in the Q4 group was increased by 1.68 times (Q4: OR 1.68, 95%CI 1.00–2.74, *P* = 0.045). Compared to the Q1 group for blood o-xylene concentration, the risk of COPD in the Q4 group was 2.19 times greater (Q4: OR 2.19, 95%CI 1.27–3.62, *P* = 0.003). The risk of developing COPD in the Q4 group versus the Q1 group for blood m-xylene concentration was 1.87 times higher (Q4: OR 1.87, 95%CI 1.20–2.88, *P* = 0.005). When comparing the Q4 group to the Q1 group for blood toluene concentration, the increased risk of COPD was 1.76 times (Q4: OR 1.76, 95%CI 1.12–2.79, *P* = 0.015). For further specific details, please see Table 3.

**Table 3:** Association of blood volatile organic compounds with chronic obstructive pulmonary disease.

Exposure	Crude model			Model 1			Model 2		
	OR	95% CI	*P*-value	OR	95% CI	*P*-value	OR	95% CI	*P*-value
Tetrachloroethene									
Q1	Reference			Reference			Reference		
Q2	0.70	0.11–2.23	0.60	0.58	0.09–1.89	0.50	0.67	0.11–2.23	0.60
Q3	0.71	0.12–2.29	0.60	0.62	0.10–2.03	0.50	0.72	0.12–2.43	0.70
Q4	0.70	0.11–2.23	0.60	0.67	0.11–2.18	0.50	0.64	0.10–2.13	0.50
Benzene									
Q1	Reference			Reference			Reference		
Q2	1.57	0.90–2.59	0.094	2.00	1.13–3.36	0.012	2.04	1.14–3.49	0.012
Q3	1.94	1.17–3.09	0.007	2.36	1.40–3.83	< 0.001	2.03	1.16–3.41	0.010
Q4	2.45	1.54–3.77	< 0.001	3.19	1.95–5.07	< 0.001	2.70	1.58–4.49	< 0.001
1,4-Dichlorobenzene									
Q1	Reference			Reference			Reference		
Q2	0.87	0.49–1.44	0.60	0.97	0.54–1.63	0.90	0.84	0.44–1.48	0.60
Q3	0.84	0.48–1.40	0.50	1.01	0.56–1.70	0.90	0.95	0.52–1.64	0.60
Q4	1.28	0.80–1.98	0.30	1.76	1.06–2.84	0.023	1.68	1.00–2.74	0.045
O-Xylene									
Q1	Reference			Reference			Reference		
Q2	1.79	1.03–2.95	0.029	1.66	0.94–2.77	0.063	1.60	0.90–2.70	0.092
Q3	1.46	0.79–2.49	0.20	1.53	0.82–2.64	0.20	1.15	0.58–2.09	0.70
Q4	2.37	1.44–3.73	< 0.001	2.52	1.51–4.04	< 0.001	2.19	1.27–3.62	0.003
M-p-Xylene									
Q1	Reference			Reference			Reference		
Q2	0.76	0.43–1.26	0.30	0.710	0.40–1.19	0.20	0.71	0.39–1.21	0.20
Q3	1.31	0.82–2.01	0.20	1.340	0.84–2.09	0.20	1.22	0.75–1.94	0.40
Q4	1.99	1.34–2.91	< 0.001	2.200	1.46–3.29	< 0.001	1.87	1.20–2.88	0.005
Toluene									
Q1	Reference			Reference			Reference		
Q2	0.90	0.56–1.43	0.70	0.94	0.58–1.51	0.80	0.98	0.60–1.61	0.90
Q3	0.74	0.45–1.21	0.20	0.72	0.43–1.17	0.20	0.75	1.44–1.24	0.30
Q4	1.67	1.12–2.52	0.01	1.99	1.31–3.05	0.001	1.76	1.12–2.79	0.015
Chloroform									
Q1	Reference			Reference			Reference		
Q2	1.17	0.72–1.83	0.50	1.150	0.70–1.82	0.60	1.16	0.96–1.88	0.60
Q3	1.39	0.85–2.18	0.20	1.310	0.80–2.07	0.30	1.48	0.89–2.37	0.11
Q4	1.00	0.60–1.60	0.90	1.020	0.60–1.64	0.90	1.14	0.67–1.85	0.60
Bromodichloromethane									
Q1									
Q2	1.37	0.61–2.66	0.40	1.440	0.63–2.84	0.30	1.43	0.62–2.88	0.40
Q3	0.36	0.06–1.16	0.20	0.410	0.07–1.33	0.20	0.48	0.08–1.56	0.30
Q4	0.87	0.30–1.93	0.80	1.040	0.63–2.35	0.90	1.15	0.40–2.65	0.80

OR, Odds ratio; Crude Model, No covariates were adjusted, Model 1 were adjusted for sex, age and race/ethnicity. Model 2 were adjusted for sex, age and race/ethnicity, poverty income ratio, BMI, educational level, drinking status. COPD, chronic obstructive pulmonary disease.

### Nonlinearity analysis using RCS

**Figure 2:**
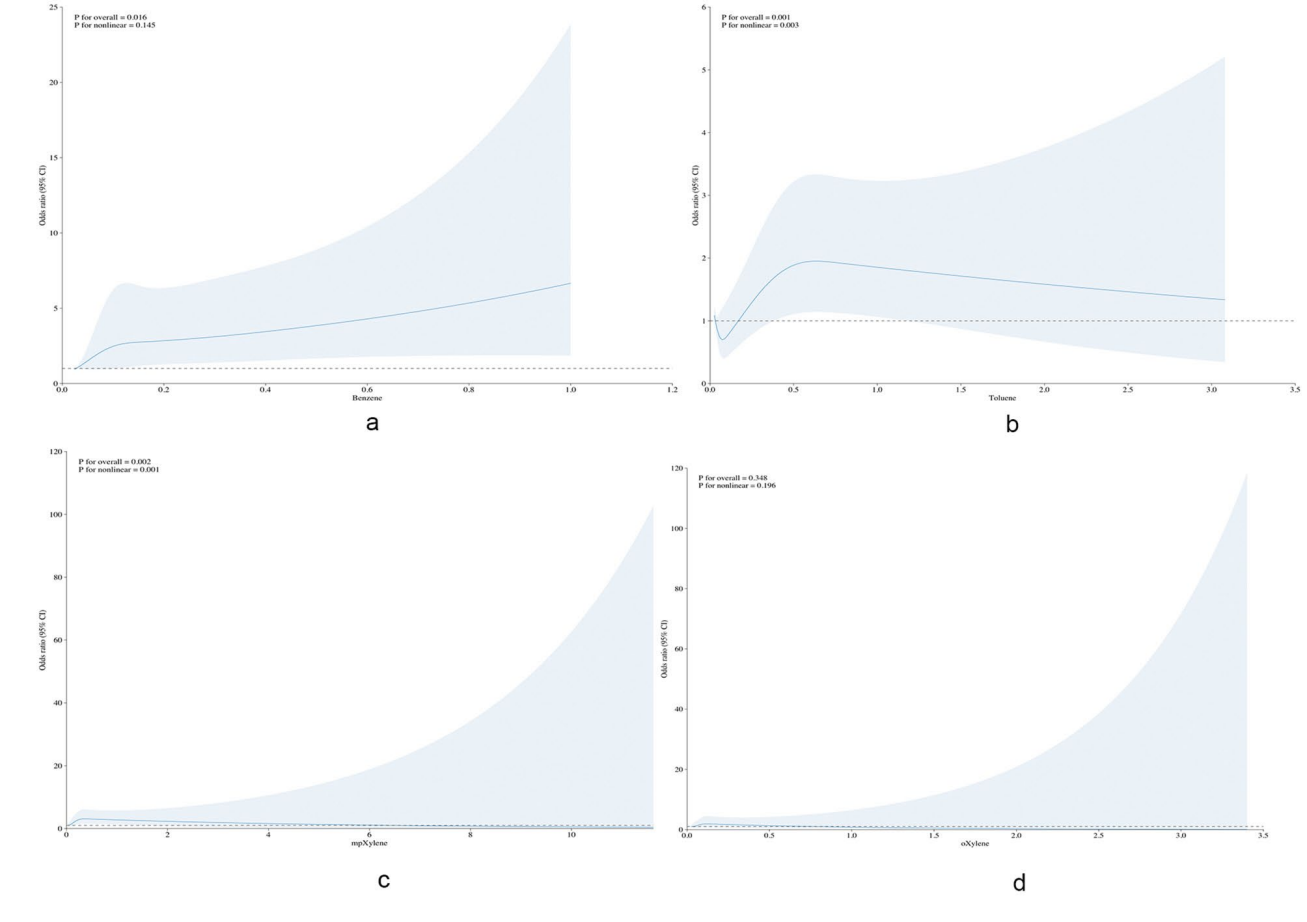
The non-linear relationship between VOCs and COPD, (**a**) Benzene, (**b**) Toluene, (**c**) M-p-Xylene, (**d**) O-Xylene. Graphs show ORs for COPD according to volatile organic compounds adjusted for sex, age, race/ethnicity, educational level, BMI, poverty income ratio, drinking status. Data were fitted by a logistic regression model, and the model was conducted with 4 knots at the 5th, 35th, 65th, 95th percentiles of volatile organic compounds (reference is the 5th percentile). Solid lines indicate ORs, and shadow shape indicate 95% CIs. OR, odds ratio; CI, confidence interval; COPD, chronic obstructive pulmonary disease; VOCs, volatile organic compounds.

We utilized RCS models to evaluate the dose–response relationship between the concentrations of Benzene, Toluene, M-p-Xylene, O-Xylene in the blood and the incidence of COPD. Knots in the model were placed at the 5th, 35th, 65th, and 95th percentiles for each volatile organic compound, using the 5th percentile as a reference. Adjustments were made for sex, age, race, education level, BMI, poverty income ratio, and drinking status. Analysis indicated that there is a positive correlation between Benzene concentration in blood and the incidence of COPD (*P* for Overall = 0.016, *P* for Nonlinear = 0.145), as shown in Fig. 2a. A significant nonlinear relationship was observed between Toluene concentration in the blood and the incidence of COPD (*P* for Overall = 0.001, *P* for Nonlinear = 0.003), as depicted in Fig. 2B. There was a significant nonlinear correlation between the concentration of M-p-Xylene in the blood and the incidence of COPD (*P* for Overall = 0.002, *P* for Nonlinear = 0.001), shown in Fig. 2c. The nonlinear relationship between the concentration of O-Xylene in the blood and the incidence of COPD was not statistically significant (*P* for Overall = 0.348, *P* for Nonlinear = 0.196), as illustrated in Fig. 2d.

### Weighted quantile sum (WQS) regression

**Figure 3:**
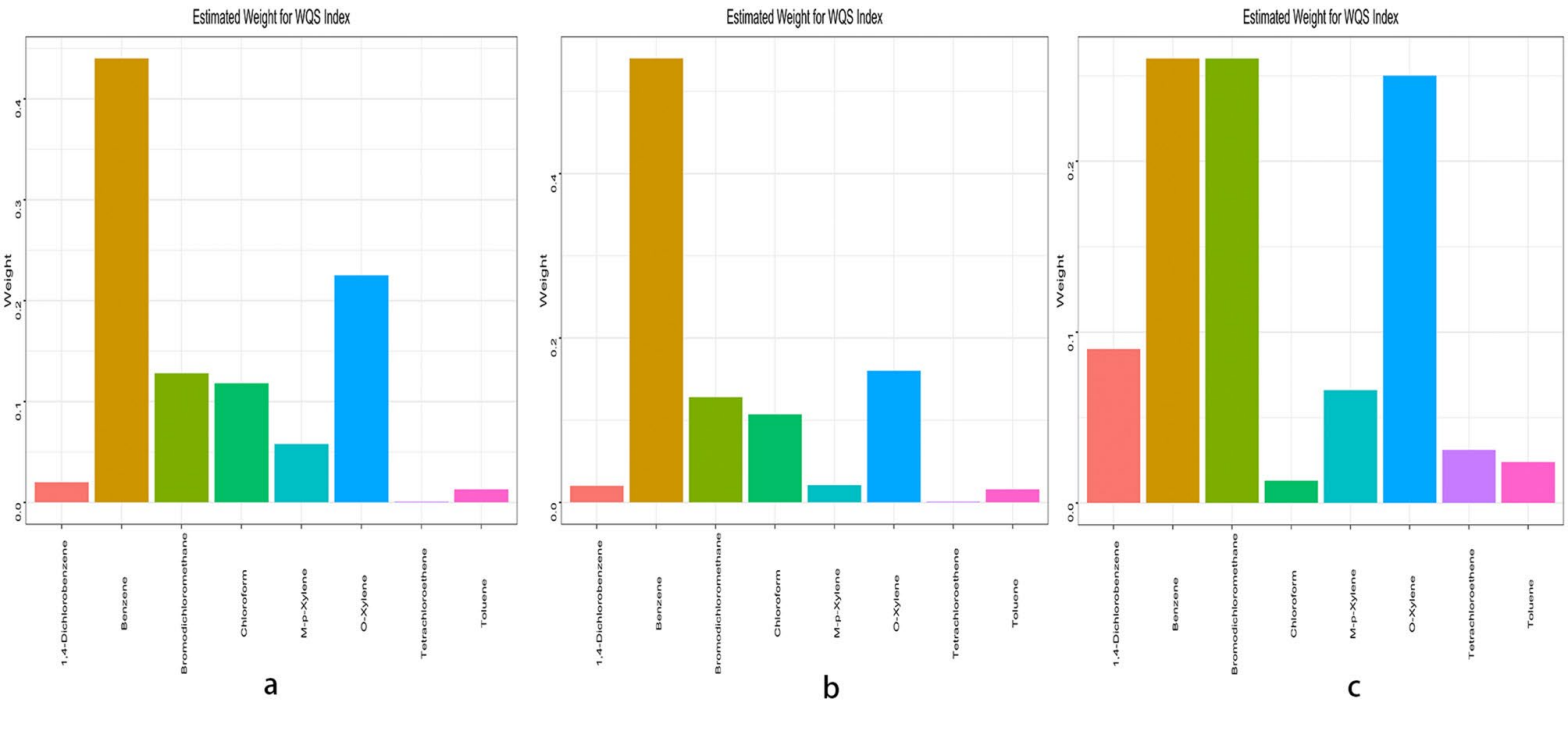
WQS model regression index weights for the COPD. (**a**) No covariates were adjusted, (**b**) Models were adjusted for sex, age and race/ethnicity. COPD, chronic obstructive pulmonary disease, (**c**) Models were adjusted for sex, age and race/ethnicity, poverty income ratio, BMI, educational level, drinking status. COPD, chronic obstructive pulmonary disease.

The WQS regression modeling was used to construct a weighted index for estimating the cumulative impact and proportional weights of Tetrachloroethene, Benzene, 1,4-Dichlorobenzene, O-Xylene, M-p-Xylene, Toluene, Chloroform, and Bromodichloromethane on COPD across three models. The results suggest: In the crude model, Benzene (43.71%) and O-Xylene (22.54%) had the highest weight percentages in blood, while Tetrachloroethene had the least weight (0.10%). For more details, please refer to Fig. 3a. In Model 1, Benzene (54.12%) and O-Xylene (16.41%) contributed the highest weight percentages in blood, with Tetrachloroethene having the lightest weight (0.15%). For specifics, see Fig. 3b. In Model 2, the highest weights in blood were attributed to Benzene (26.54%) and Bromodichloromethane (26.27%), while Chloroform had the lowest weight (0.13%). Details are presented in Fig. 3c.

### Machine learning models performance

**Figure 4:**
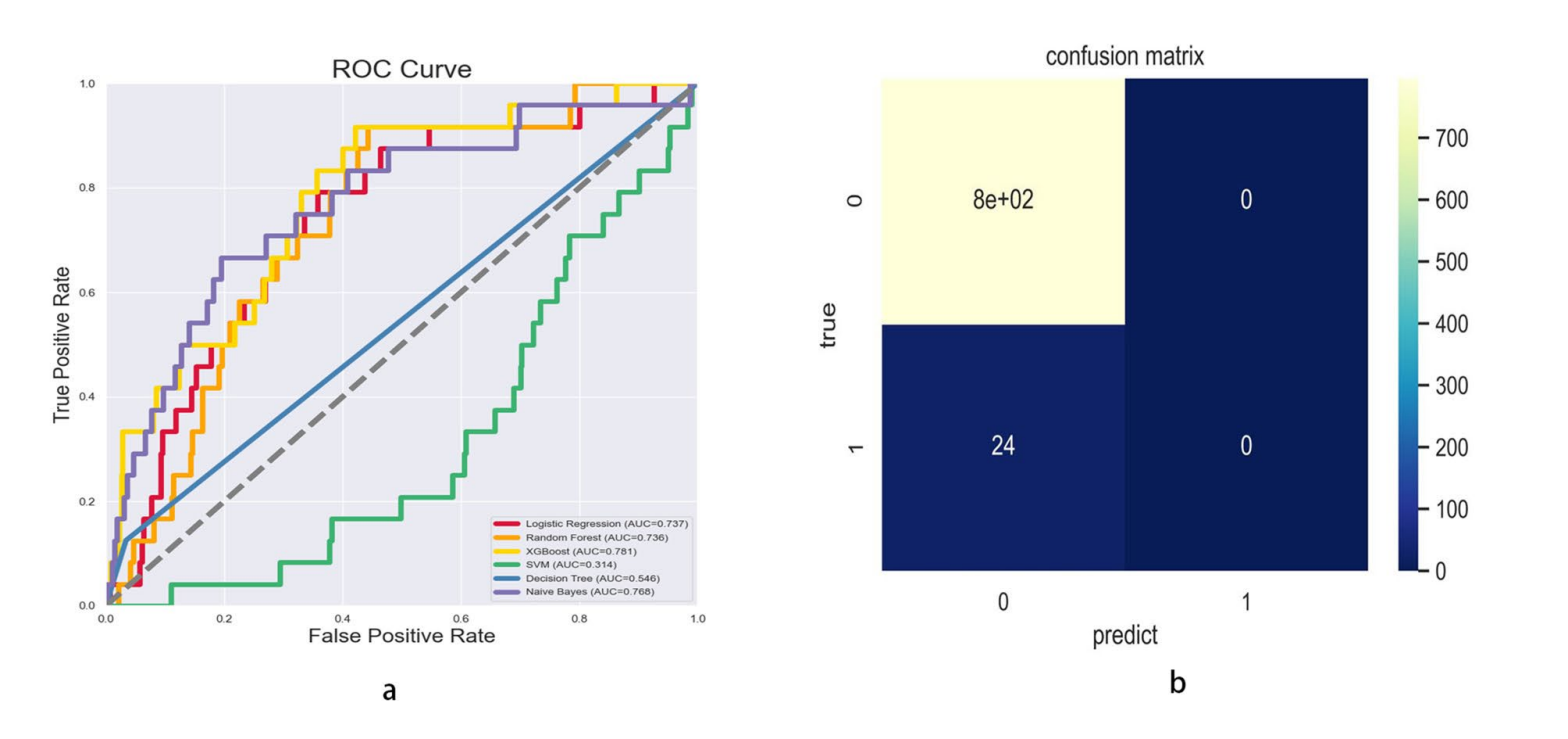
(**a**) ROC curves of six machine learning models. (**b**) Confusion matrix analysis of XGBoost model.

This study evaluated the predictive ability of six machine learning models by drawing ROC curves and calculating AUC values. The AUC values of the training sets of the six machine learning models ranged from 0.314 to 0.781, among which, the XGBoost model had the highest AUC value (AUC = 0.781) (Fig. 4a). In the evaluation indicators of the XGBoost model, the accuracy was 97.09%, the precision was 100.00%, the recall was 97.09%, and the F1-Score was 98.52%. More details can be seen in Fig. 4b. Therefore, we can conclude that the XGBoost model performed the best in predicting the occurrence of COPD.

### The dual-pollutant model analysis

In the dual-pollutant model, an average increase of 1 ng/ml in Benzene, O-Xylene, and M-p-Xylene was associated with a 88% (95% CI, 1.30–2.70), 51% (95% CI, 1.02–2.22), and 58% (95% CI, 1.15–2.17) higher incidence of COPD, respectively. No significant associations were observed between Tetrachloroethene, 1,4-Dichlorobenzene, Toluene, Chloroform, Bromodichloromethane, and COPD. Further details are provided in Table 4.

**Table 4:** Association between urinary VOCs concentrations and chronic obstructive pulmonary disease (COPD) using a dual pollutant model.

VOCs (urine, ng/ml)	OR	Lower CI	Upper CI	*P* value
Tetrachloroethene	0.70	0.27	1.86	0.477
Benzene	1.88	1.30	2.70	< 0.001
1,4-Dichlorobenzene	1.31	0.91	1.88	0.153
O-Xylene	1.51	1.02	2.22	0.038
M-p-Xylene	1.58	1.15	2.17	0.005
Toluene	1.18	0.86	1.62	0.304
Chloroform	1.24	0.88	1.75	0.210
Bromodichloromethane	0.83	0.40	1.75	0.629

*OR*, Odds ratio.

Adjusted for age, BMI, PIR, race, and drinking status.

## Discussion

In this nationally representative cross-sectional study, we found strong evidence that higher blood concentrations of several VOCs, including benzene, toluene, ethylbenzene and xylenes, were significantly associated with increased odds of COPD in American adults. Our flexible nonlinear exposure–response analyses using RCS models further revealed that the relationships between blood benzene, toluene and xylene levels with COPD prevalence were nonlinear and non-monotonic, challenging the linear no-threshold model commonly assumed for environmental exposures. These complex dose–response patterns warrant future investigations to elucidate the underlying biological mechanisms. Moreover, WQS regression suggested varying effect sizes of different VOCs on COPD, with benzene and o-xylene having the largest weights. This highlights the importance of examining mixtures of VOCs, rather than individual chemicals in isolation, when assessing the impact of VOCs exposure on respiratory health. Besides, this study introduced a dual-pollutant model to investigate the combined exposure of multiple VOCs and their relationship with COPD. This model not only considers the individual effects of single compounds but also evaluates the combined impact of interactions between different compounds on the risk of COPD. This study found that the XGBoost algorithm performs best in diagnosing COPD. The XGBoost model, as an excellent gradient boosting machine learning model, is widely used in multiple medical fields such as chronic kidney disease^18^, and bone metastasis of prostate cancer^19^. Based on the structure of the decision tree model, it optimizes the number of decision trees it contains, reduces the gradients of model errors, prevents overfitting of the model in order to improve the overall accuracy of the prediction model. Our study provides novel and compelling evidence that environmental exposure to VOCs, especially aromatic hydrocarbons like benzene and toluene, may play a significant role in COPD pathogenesis in the general population.

Several potential biological mechanisms may underlie the observed associations between elevated blood VOC levels and increased COPD prevalence. Previous toxicological studies have demonstrated that many VOCs such as benzene, toluene and xylenes can induce significant oxidative stress and inflammation in the lungs through the generation of reactive oxygen species, lipid peroxidation, and pro-inflammatory cytokines^20,21^. The resulting oxidative damage and chronic inflammation are recognized as major drivers in the pathogenesis of COPD^22^. In particular, benzene and its metabolites have been shown to stimulate the release of inflammatory cytokines like IL-6 and TNF-αin lung epithelial cells and alveolar macrophages^20,23^. Toluene can also cause persistent lung inflammation and airway remodeling changes in animal models^20^. Moreover, animal studies have revealed that VOCs exposure could directly impair lung function through reducing mucociliary clearance, disrupting cilia structure, surfactant production and clara cell toxicity^24–26^. Some VOCs including benzene and xylenes are also associated with increased risks of respiratory infections based on epidemiological studies^27,28^, which can exacerbate COPD progression. Additionally, VOCs may interact synergistically with other pollutants found in cigarette smoke, such as carbon monoxide, cyanides, polycyclic aromatic hydrocarbons, and ozone, enhancing smoke-induced injuries and increasing susceptibility to COPD^29,30^. The complex mechanisms likely involve oxidative stress, inflammation, lung epithelial injury, ciliary dysfunction, immunotoxicity, and susceptibility to infections. Further toxicological and experimental studies in animal and cellular models are warranted to fully elucidate the biological mechanisms underlying the impact of VOCs exposure on COPD pathogenesis.

Our study has important public health implications. To our knowledge, it represents one of the first studies to examine the relationship between exposure to VOCs mixtures and COPD prevalence in a nationally representative population-based sample. The findings provide compelling evidence that exposure to environmental VOC pollutants, especially aromatic hydrocarbons like benzene and toluene, may contribute significantly to COPD development in the general population. The detailed exposure–response relationships further suggest complex non-linear and non-monotonic patterns underlying VOC-associated COPD risks, challenging the linear no-threshold model commonly assumed. These data can inform the monitoring and control of VOCs emissions and exposures to mitigate COPD morbidity. Our results also underscore the importance of examining cumulative effects of VOC mixtures, rather than individual chemicals. The varying weights of different VOCs from WQS regression may aid priority setting in the regulation and abatement of volatile organic pollutants to reduce COPD incidence. Overall, this study enhances our understanding of environmental risk factors for COPD beyond smoking and provides a basis for future investigation on VOC-COPD linkages. Translating these findings may lead to improved policies to better control VOC emissions and protect susceptible populations from avoidable COPD burdens.

There were several innovative methodological strengths in our study. First, we utilized flexible nonlinear exposure–response models including restricted cubic splines to assess potential non-linear relationships between VOCs exposure and COPD risk. This advanced approach avoids assuming linearity and provides more valid risk estimations compared to traditional linear models. Second, we applied the WQS regression to examine the cumulative effects of multiple VOCs and quantify the contribution of each chemical. The application of the dual pollution model takes into account the simultaneous exposure to multiple VOCs and their combined impact on COPD. These two technologies overcome the limitations of conventional single-pollutant models and captures potential interactions within VOC mixtures. Third, the large nationally representative NHANES sample ensures high generalizability of our findings to the US adult population. The robust design and adequate sample size also help enhance result validity and precision. Furthermore, the XGBoost model shows promise for extensive application in future research to aid clinicians in making effective clinical decisions and conducting more efficient early screening and diagnosis for COPD patients. Overall, this study integrates state-of-the-art statistical methodologies and a powerful population-based dataset to provide novel and compelling evidence supporting the role of environmental VOCs exposure in COPD pathogenesis. Our analytic strategies can serve as a model for future environmental epidemiology studies on examining complex effects of chemical mixtures.

Our study has several limitations worth noting. First, the cross-sectional design precludes causal inference and cannot determine temporal relationships between VOCs exposure and COPD onset. In our study, we used the NHANES database, which provides data on subjects’ VOC exposure over a specific period. However, these data capture only short-term exposure, lacking details on exposure duration and information from longitudinal cohorts. As a result, we could not accurately determine the length of exposure or whether subjects were continuously or acutely exposed to VOCs, nor could we conduct large cohort studies for further validation. To address this issue, we recommend incorporating longitudinal follow-up studies in future research designs to obtain long-term exposure data for participants. Additionally, combining NHANES data with other long-term exposure studies for comprehensive analysis of chronic exposure impacts is advisable. Furthermore, considering the use of biomarkers that reflect long-term exposure can help compensate for the limitations of the database. Second, although we adjusted for an array of covariates, residual confounding from unmeasured factors cannot be ruled out given the observational nature. Considering the limitations of the NHANES database, we were unable to account for crucial influencing factors such as temperature and humidity in this study. Future research should integrate local environmental monitoring data, such as meteorological data, to more comprehensively analyze VOC exposure. Additionally, using environmental exposure models to predict VOC levels under varying temperature and humidity conditions and correlating these predictions with health data is recommended. Third, some VOCs like chloroform had relatively low detection rates, which may underestimate their true associations with COPD. Additionally, we did not evaluate specific VOC exposure sources or have data on subjective asthma history. Further studies with comprehensive VOC exposure assessments are needed to validate and extend our findings.

Notwithstanding these limitations, this study provides novel and important evidence underscoring the potential effects of environmental VOC exposure on COPD risks in the general population. In summary, by incorporating longitudinal studies, integrating environmental data, and utilizing biomarkers, we can gain a more comprehensive understanding of the relationship between VOC exposure and COPD. This approach will provide more reliable data support for future research and contribute to better protection of public health.

## Conclusion

In conclusion, this population-based study provides epidemiological evidence that exposure to VOCs, especially benzene and toluene, is significantly associated with increased prevalence of COPD among US adults. The relationships are nonlinear and VOC mixtures may jointly contribute to COPD risks. These data suggest that environmental VOCs exposure may be a novel risk factor involved in COPD pathogenesis. Further research is warranted to confirm our findings and explore the underlying mechanisms. Specifically, large prospective cohort studies are needed to establish temporal relationships and derive causal inferences. Detailed exposure assessments should be conducted to identify major VOC sources and determine their relative contributions. The impact of VOCs on COPD exacerbations and disease progression also merits investigation. Moreover, validating the results in other populations with different VOC exposure patterns would help generalize the findings. In summary, this study takes an important first step in elucidating the potential effects of environmental VOCs on COPD development and underscores the necessity for a multifaceted research agenda to corroborate, extend and translate our results. These future efforts would provide a strong basis for implementing policies to better control VOC emissions and mitigate associated COPD burdens.

## Data Availability

The data supported this research can be downloaded here: https://www.cdc.gov/nchs/nhanes/index.htm.
